# Collagen supplementation augments changes in patellar tendon properties in female soccer players

**DOI:** 10.3389/fphys.2023.1089971

**Published:** 2023-01-26

**Authors:** Joonsung Lee, Josh E. Bridge, David R. Clark, Claire E. Stewart, Robert M. Erskine

**Affiliations:** ^1^ School of Sport and Exercise Sciences, Liverpool John Moores University, Liverpool, United Kingdom; ^2^ Institute of Sport, Exercise and Health, University College London, London, United Kingdom

**Keywords:** football (soccer), vitamin C (ascorbate), resistance training (strength), Young’s modulus (elastic modulus), elite

## Abstract

We investigated the effect of collagen hydrolysate supplementation on changes in patellar tendon (PT) properties after 10 weeks’ training in female soccer players from a Football Association Women’s Super League Under 21 s squad. We pair-matched *n* = 17 players (age: 17 ± 0.9 years; height: 1.66 ± 0.06 m; mass: 58.8 ± 8.1 kg) for baseline knee extension (KE) maximum isometric voluntary contraction (MIVC) torque, age, height, and body mass, and randomly assigned them to collagen (COL) or placebo (PLA) groups (COL *n* = 8, PLA *n* = 9). Participants consumed 30 g collagen hydrolysate supplementation or energy-matched PLA (36.5 g maltodextrin, 8.4 g fructose) and plus both groups consumed 500 mg vitamin C, after each training session, which comprised bodyweight strength-, plyometric- and/or pitch-based exercise 3 days/week for 10 weeks in-season. We assessed KE MIVC torque, *vastus lateralis* muscle thickness and PT properties using isokinetic dynamometry and ultrasonography before and after 10 weeks’ soccer training. KE MIVC torque, muscle thickness and tendon cross-sectional area did not change after training in either group. However, COL increased PT stiffness [COL, +18.0 ± 12.2% (*d* = 1.11) vs. PLA, +5.1 ± 10.4% (*d* = 0.23), *p* = 0.049] and Young’s modulus [COL, +17.3 ± 11.9% (*d* = 1.21) vs. PLA, +4.8 ± 10.3% (*d* = 0.23), *p* = 0.035] more than PLA. Thus, 10 weeks’ in-season soccer training with COL increased PT mechanical and material properties more than soccer training alone in high-level female soccer players. Future studies should investigate if collagen hydrolysate supplementation can improve specific aspects of female soccer performance requiring rapid transference of force, and if it can help mitigate injury risk in this under-researched population.

## Introduction

Soft tissue injury (including collagenous tissues, such as skeletal muscle, tendon and ligament) is the most common injury type in women’s soccer (López-Valenciano et al., 2021; Mayhew et al., 2021), and is two-to-five times more common in female than in male soccer players (Waldén et al., 2011; Larruskain et al., 2018). Indeed, female players suffer a higher proportion of quadriceps muscle strains, knee ligament injuries and anterior cruciate ligament (ACL) ruptures than male players (Larruskain et al., 2018). Although this sex difference in injury risk is likely multifactorial, ACL injury specifically can be partially ascribed to greater knee joint laxity in women than men (Rozzi et al., 1999). This sex-difference is reflected in women having a more compliant patellar tendon than men (Onambele et al., 2007; Hicks et al., 2013), which may be linked to higher circulating levels of estrogen in women (Hansen et al., 2013). As tendon and ligament comprise ∼80% type I collagen (Frank, 2004; Kjaer, 2004), and circulating levels of estrogen fluctuate during the menstrual cycle in eumenorrheic women, this may explain the higher incidence of muscle and tendon injuries in the late follicular phase of the menstrual cycle, i.e., when circulating estrogen concentration peaks (Martin et al., 2021). Another injury risk factor in women’s soccer is muscle weakness (Crossley et al., 2020), so it follows that incorporating resistance exercise into a soccer training program should increase strength, thereby reducing injury risk. However, despite the aforementioned sex differences in tendon properties and soft tissue injury risk, tendon properties have not been investigated in female soccer players, so it is not known to what extent their tendons can adapt to strength training.

Traditional strength (Reeves et al., 2003; Kongsgaard et al., 2007; Seynnes et al., 2009) or plyometric (Foure et al., 2010) training is known to increase both stiffness and Young’s modulus of human tendon *in vivo*. As there is a linear relationship between tendon stiffness and ultimate stress (LaCroix et al., 2013), a stiffer tendon has a greater loading capacity, thus potentially reducing tendon injury risk during periods of high mechanical load, e.g. during soccer training/match play. Furthermore, a stiffer tendon is associated with a faster rate of torque development (Bojsen-Møller et al., 2005), which is also linked to greater lower-limb strength and power outputs (Bojsen-Møller et al., 2005; Wdowski et al., 2022). Thus, strength training could have multiple benefits for soccer players, with changes in tendon properties likely due to the cumulative remodeling that occurs after each bout of exercise. Indeed, resistance-type exercise increases human patellar tendon collagen fractional synthetic rate (Miller et al., 2005), while endurance-type exercise increases circulating concentration of procollagen type 1 N-terminal propeptide (P1NP, a biomarker of collagen synthesis) in humans (Langberg et al., 1999; Langberg et al., 2001). Newly synthesized procollagen molecules then undergo post-translational modifications, for which the presence of vitamin C is an essential co-factor during collagen synthesis (Murad et al., 1981), transport and assembly into tendon (Canty and Kadler, 2005).

Concomitant increases in biomarkers of collagen synthesis and tendon collagen content after 2 months’ strength training, followed by an increase in tendon stiffness with a further month’s training, suggest that augmented tendon collagen synthesis and content are necessary to increase Achilles tendon mechanical properties in response to overloading (Kubo et al., 2012). Moreover, collagen synthesis after a single bout of exercise in humans appears to be further augmented with the addition of exogenous vitamin C-enriched collagen in a dose-response manner, i.e., 15 g gelatine increases serum P1NP concentration by more than two-fold compared to 5 g and 0 g (Shaw et al., 2017). Thus, further augmentation of collagen synthesis *via* collagen supplementation may cause even greater changes in tendon mechanical and material properties following a period of chronic exercise training. In support of this hypothesis, strength training with 15 g collagen supplementation has been shown to cause greater accretion of lean mass in young, healthy men (Kirmse et al., 2019). Furthermore, short-term power training with 20 g collagen supplementation appears to have a beneficial effect on the rate of force development in young male athletes (Lis et al., 2021), which suggests collagen supplementation may enhance the stiffening of the muscle-tendon unit that occurs with strength training. Interestingly, a recent study found that just 5 g collagen supplementation with strength training in young men induced greater hypertrophy of the Achilles tendon and gastrocnemius muscle than strength training alone, although tendon stiffness was not affected (Jerger et al., 2022). It is not known, however, if collagen supplementation can affect the patellar tendon in a similar manner, or whether the interaction of a larger dose of collagen with soccer-specific training can elicit greater changes in patellar tendon properties in high-level female soccer players, which would have implications for mitigating soft tissue injury risk in this population.

Therefore, the aim of the current study was to compare the effect of 10 weeks’ soccer training (incorporating strength-, plyometric- and pitch-based training) with or without collagen supplementation on changes in patellar tendon properties in female soccer players. We hypothesized that 30 g collagen hydrolysate and 500 mg vitamin C ingested immediately after each training session (three times per week) would confer changes in patellar tendon mechanical and material properties compared to no changes following soccer training alone. As strength training with collagen supplementation has recently been shown to enhance fat-free mass in healthy young men (Kirmse et al., 2019), a secondary aim was to investigate the effect of soccer training and collagen supplementation on *vastus lateralis* muscle thickness and subcutaneous adipose tissue thickness.

## Materials and methods

### Experimental overview

The study recruited high-level female soccer players from the ‘Under 21s’ squad of a Football Association (FA) Women’s Super League Academy Club and the study design was a single-blind, randomized controlled trial. Due to the study taking place during the national lockdown associated with the COVID-19 pandemic, the lead researcher was required to be unblinded to participant group allocation (collagen, COL, or placebo, PLA) from the outset of the study. This was to ensure the correct supplements were consumed by the participants (due to a national lockdown, no other researchers or technicians were available to prepare the supplements). Crucially, however, all participants were blinded to group allocation, plus the strength and conditioning coaches responsible for undertaking all training sessions were blinded to participant group allocation.

All participants attended the laboratory for muscle-tendon assessments before and after 10 weeks’ soccer training. The training took place during the 2020–21 in-season and comprised a combination of lower-limb plyometric, bodyweight strength and pitch-based soccer training. All muscle-tendon measurements were performed on the right leg in the following order: maximal isometric and isokinetic strength of the knee extensors and flexors (antagonist muscle activation was measured using surface electromyography [EMG]), thickness of the *vastus lateralis* (VL) muscle and its subcutaneous adipose tissue (SAT), and morphological, mechanical and material properties of the patellar tendon were measured *via* a combination of ultrasonography and isokinetic dynamometry (IKD). All tests took place between 09:00 and 17:00 and the pre/post-training tests were performed at the same time of day for each participant to avoid potential diurnal effects on intra-individual pre/post-training changes (Onambele-Pearson and Pearson, 2007). Further, participants were instructed not to participate in strenuous physical activity, not to consume alcohol or caffeine in the 24 h prior to testing. Due to the busy training and match schedule during in-season, staggered testing was performed within 2 weeks. Participants were then pair-matched according to age, height, body mass, maximum knee extensor strength and use (or not) of hormonal contraception, and then randomly assigned to one of two groups (COL or PLA) using block randomization. Participants were instructed to ingest their respective supplement three times a week with training for the 10-week period. Each participant completed all 30 training sessions, and ingested all of their 30 respective supplements (supervised by a member of the research team). Post-training assessments were performed within three-to-five days after the final supplemented training session.

### Participants

*A priori sample size estimation:* A minimal sample size was estimated prior to conducting the study with G*Power software (version 3.1.9.6, Heinrich-Heine-Universität Düsseldorf, Düsseldorf, Germany). Briefly, the estimation was performed using the effect size (*η*_*p*_^2^ = 0.163) from the time (pre and post 12 weeks’ strength training) × group (COL vs. PLA) interaction (*p* = 0.002) regarding the change in fat-free mass in the study by (Kirmse et al., 2019). The results from our *a priori* power calculation deemed a minimum of 14 participants was necessary to detect an effect of COL vs. PLA (two-way analysis of variance (ANOVA); α: 0.05; power: 0.80).

Twenty-one female soccer players from the ‘Under 21s’ squad volunteered to take part in this study after providing written informed consent. However, three players were unable to be scheduled for baseline testing and, in the week before post-training testing, one participant suffered an injury unrelated to the study and was unable to complete the testing. Following random allocation into COL and PLA (after pair-matching, as described above), characteristics of the 17 participants, who completed the study are displayed in Table 1. COL comprised one goalkeeper, three defenders, two midfielders and two forwards, while PLA comprised one goalkeeper, three defenders, four midfielders and one forward.

**TABLE 1:** Participant baseline characteristics. Data are mean ± SD.

Variable	COL (*n* = 8)	PLA (*n* = 9)
Age (years)	17.0 ± 0.8	16.8 ± 1.0
Height (m)	1.65 ± 0.08	1.67 ± 0.04
Body mass (kg)	62.0 ± 10.7	57.4 ± 6.2
ISO KE MVC (N·m)	185 ± 41	183 ± 48
ISO KF MVC (N·m)	84.2 ± 22.0	80.9 ± 20.2
CON KE MVC (N·m)	138 ± 31	131 ± 29

*COL*, collagen group; *PLA*, placebo group; *ISO*, isometric; *KE*, knee extension; *MVC*, maximal voluntary contraction torque; *KF*, knee flexion; *CON*, concentric. There were no differences between COL, and PLA (all *p* > 0.05).

Exclusion criteria for all participants included history of lower limb muscle/tendon injuries in the 6 months prior to the start of the study; consumption of nutritional supplementation that purportedly affects muscle-tendon adaptation or recovery (e.g., protein powder, vitamin C, collagen); being vegan or vegetarian (due to the mammalian source of collagen); previous anterior cruciate ligament injury where the patellar tendon was used as a graft; age <16 years or >39 years. All participants provided written informed consent prior to commencing the study, which was approved by Liverpool John Moores University Research Ethics Committee and complied with the Declaration of Helsinki.

### Identification of menstrual cycle phase and hormonal contraceptive use

During the pre-testing, participants were asked to complete a questionnaire in order to report which menstrual cycle (MC) phase they were tested in. Each participant’s MC was estimated using calendar-based counting, which requires the self-reported date of the onset of menses, number of days’ menstruation, and length of MC. The MC phases specified here were defined as “early follicular,” “late follicular,” “early luteal,” and “late luteal.” Days of follicular and luteal phases with different MC length were calculated based on the regression equation from (McIntosh et al., 1980). Also, hormonal contraceptive use was identified *via* the questionnaire to provide more detailed participant characteristics.

### Training period

The in-season training program detailed here did not deviate from the athletes’ habitual soccer training program. Participants performed four training sessions (Monday, Tuesday, Friday and Saturday) and one competitive match (Wednesday) per week, and nutritional supplementation was consumed on three of those sessions every week for 10 weeks. A typical microcycle with nutritional supplementation was Monday (pitch-based session followed by lower-limb bodyweight strength exercises), Tuesday (pitch-based session followed by lower-limb plyometric exercises), and Friday (pitch-based session). An additional pitch-based session was conducted on Saturday, which was only used as a supplementation day if participants missed one of their regular supplementation days or the match day. The volume of plyometric and bodyweight strength training was progressively increased on a weekly basis. Detailed training programs for lower-limb bodyweight strength and plyometric exercises are presented in Supplementary Table S1. Individual training load during the pitch-based sessions for 10 weeks was measured using a global positioning system device (Apex, STATSports, Newry, Northern Ireland). Total running (12.2–19.1 km h^−1^) and sprinting (>19.4 km h^−1^) (Dwyer and Gabbett, 2012) distance are presented in Supplementary Table S2.

### Nutritional intervention

Due to the high standard of athletes participating in this study, all supplements were required to be “Informed Sport” certified as having been tested by LGC Group’s anti-doping laboratory for contamination with banned substances. Participants in COL received 90 mL “Collagen Liquid” (GBR Nutrition, London, United Kingdom), which contained 30 g collagen hydrolysate, dextrose monohydrate, fructose, flavouring (mango and passion fruit), stabilisers (potassium sorbate and sodium benzoate), sweetener (sucralose), citric acid and water, and comprised 180 kcal. Participants in PLA received 49.3 g “Tropical” flavour “GO Electrolyte” (Science in Sport, London, United Kingdom), which contained 36.5 g maltodextrin, 8.4 g fructose, citric acid, electrolytes (sodium chloride, calcium lactate, potassium chloride, sodium citrate, magnesium citrate), natural flavouring sweetener (aspartame) and phenylalanine (source), and comprised 180 kcal. Thus, PLA was calorie- and taste-matched with the COL supplement. Each supplement was mixed with water to create a total volume of 250 mL, and all participants in both groups were given a 500 mg vitamin C tablet (Elite Vitamin C, Healthspan, Guernsey, United Kingdom) to consume immediately after consuming the drink. Participants consumed their supplements in entirety immediately after each training session (i.e., three times a week for 10 weeks) under the supervision of the strength and conditioning coach, who was blinded to group allocation. All drinks were provided in opaque bottles and, together with the taste-matching and equal volume of drink, this ensured participants were blinded to their allocated group for the entirety of the study. The number of nutritional supplements that participants consumed during the different types of training session is shown in Supplementary Table S3.

### Habitual dietary behavior and anthropometry

Participants’ height (SECA, model-217, Hamburg, Germany) and body mass (SECA, model-875, Hamburg, Germany) were measured to the nearest 0.1 cm and 0.1 kg, respectively. Participants were asked to record their habitual dietary behavior using a food and drink diary for 3 days (Thursday to Saturday) during the baseline testing period. This aspect of the study was completed by *n* = 14 (Table 2). Records were analyzed with Nutritics professional dietary analysis software (version 5.09, Nutritics Ltd. Co. Dublin, Ireland) to obtain total energy, macro- and micronutrient composition. All daily nutritional composition data were presented as absolute and relative (normalized to body mass) values. “Total intake” was calculated as the sum of habitual intake and nutritional supplementation used in this study. Thus, the total COL supplementation on training days was 30 g d^−1^, and when averaged across training and non-training days, the COL supplement increased protein intake by 12.9 g d^−1^, vitamin C intake by 214 mg d^−1^ and energy intake by 77.1 kcal d^−1^. Each PLA supplement contained 44.9 g maltodextrin/fructose and, when averaged across training and non-training days, increased carbohydrate intake by 19.2 g d^−1^, vitamin C intake by 214 mg d^−1^ and energy intake by 77.1 kcal d^−1^.

**TABLE 2:** Energy, macronutrient, and micronutrient intake during the pre-training assessment period. Data are mean ± SD.

Nutritional composition	COL (*n* = 6)	PLA (*n* = 8)	*t*-test, *p*
*Energy intake*			
Habitual intake (kcal·d^−1^)	1634 ± 162	1553 ± 200	0.433
Total intake (kcal·d^−1^)	1711 ± 162	1630 ± 200	0.433
*Carbohydrate intake*			
Habitual intake (g·d^−1^)	204 ± 37	187 ± 33	0.378
Habitual intake (g·kg·d^−1^)	3.2 ± 0.7	3.3 ± 0.9	0.772
Total intake (g·d^−1^)	204 ± 55	206 ± 33	0.916
Total intake (g·kg·d^−1^)	3.2 ± 0.7	3.7 ± 1.0	0.344
*Protein intake*			
Habitual intake (g·d^−1^)	79.7 ± 9.0	77.3 ± 14.7	0.733
Habitual intake (g·kg·d^−1^)	1.2 ± 0.1	1.4 ± 0.3	0.373
Total intake (g·d^−1^)	92.5 ± 9.0^a^	77.3 ± 14.7	0.046
Total intake (g·kg·d^−1^)	1.4 ± 0.1	1.4 ± 0.3	0.615
*Fat intake*			
Habitual intake (g·d^−1^)	55.6 ± 6.6	56.0 ± 11.3	0.930
Habitual intake (g·kg·d^−1^)	0.9 ± 0.1	1.0 ± 0.2	0.214
*Vitamin C intake*			
Habitual intake (mg·d^−1^)	76.0 ± 26.8	96.6 ± 42.9	0.323
Habitual intake (mg·kg·d^−1^)	1.2 ± 0.5	1.7 ± 0.9	0.183
Total intake (mg·d^−1^)	290 ± 27	311 ± 43	0.320
Total intake (mg·kg·d^−1^)	4.5 ± 0.7	5.5 ± 1.3	0.103

^a^ Different from PLA (*p* < 0.05).

### Knee extensor and flexor maximal voluntary contraction

Participants performed isometric and isokinetic knee extension (KE) and isometric knee flexion (KF) maximal voluntary contractions (MVCs) on an IKD (Humac Norm, Computer Sports Medicine Inc. Stoughton, United States) to determine KE and KF MVC torque. Knee and hip joint angles were set at 90° (0° = full knee extension) and 85° (180° = supine), respectively, and movement was restricted with the use of inextensible waist, chest, and thigh straps. Participants performed a standardized warm up comprising 10 repetitions of KE and KF (60 s^−1^; full range of motion) by gradually increasing intensity from sub-maximal to maximal for preconditioning the tendon (Maganaris, 2003). Participants then performed three concentric KE MVCs at 60 s^−1^ (full range of motion) interspersed with 5-s rest, and the highest of the three attempts was used for subsequent analysis. After 5 minutes’ rest, participants performed three isometric KE and KF MVCs (each lasting 3 seconds), alternating between KE and KF every 30 s. If the highest MVC differed from the next highest by >5%, a further attempt was made to ensure the highest MVC was achieved (which was generally attained within three attempts). However, in the event that the highest KE MVC performed during the ramped KE isometric MVC (RMVC, for more details see below), then this value was used to represent KE isometric MVC instead. The torque signal was interfaced with an analogue-to-digital converter (MP150 Biopac Systems Inc. Santa Barbara, United States), sampled at 2 kHz with a PC using data acquisition software (AcqKnowledge version 5.1, Biopac Systems Inc.) and low-pass filtered (10 Hz edge frequency) offline.

### Morphological, mechanical, and material tendon properties

#### Antagonist muscle co-activation

To estimate the extent of antagonist (hamstring) muscle co-activation during a KE RMVC, the EMG activity of the biceps femoris long head (BFlh) was recorded, which represents the knee flexor muscle group (Kellis and Baltzopoulos, 1999). The BFlh was identified *via* palpation during a submaximal knee flexion in the anatomical position. After preparation of the skin surface (shaving, skin abrasion with a sand paper and cleaning the skin with an alcohol wipe), two bipolar Ag-AgCl surface electrodes (Neuroline, Medoicotest, Rugmarken, Denmark) with 20 mm inter-electrode distance were placed on the sagittal axis of the BFlh. The location of surface electrodes was on the distal third of the BFlh length according to SENIAM guidelines (Hermens et al., 2000) and one reference electrode was placed on the lateral tibial condyle. The EMG signal was band-pass filtered (10–500 Hz) and the root mean square (RMS) was calculated every 300 ms. Assuming a linear relationship between BFlh RMS EMG and KF MVC torque output (Kellis and Baltzopoulos, 1997; Reeves et al., 2004), KF co-activation torque during increment of RMVC and relaxation was calculated as:

$$\frac{BFlh\;RMS\;EMG\;during\;KE\;RMVC}{BFlh\;RMS\;EMG\;during\;KF\;MVC}\times peak\;KF\;MVC\;torque$$

The antagonist co-activation torque was subsequently added to the KE torque at the relevant RMVC to provide the net KE MVC torque. To estimate tendon force, the net KE torque was divided by the patellar tendon moment arm at 90° knee flexion, which was estimated from each participant’s femur length (Visser et al., 1990).

#### Patellar tendon cross-sectional area

Participants were seated on the IKD in the resting state, with the knee secured at 90°. A 4-cm wide 5–18 MHz linear probe (Philips EPIQ seven Ultrasound System, Bothel, United States) was placed sagittally on the skin overlying the patellar tendon to measure resting tendon length, defined as the distance between the patella apex and the tibial tuberosity. Using a permanent marker pen, locations corresponding to 25%, 50%, and 75% tendon length were marked on the skin and the ultrasound probe was placed on each location in the axial plane to image the tendon cross-sectional area (CSA).

#### Measurement of tendon elongation

Participants remained seated on the IKD and a 2-mm wide strip of surgical tape (3M, Neuss, Germany) was placed on the skin, transversely over the patellar tendon at ∼50% tendon length, to act as an echo-absorptive marker that would be visible in the subsequent tendon elongation video scans. This was to ensure the transducer did not move with respect to the tendon throughout the RMVC but, if it did, the RMVC was repeated. Prior to performing the RMVC, participants competed two to three submaximal ramped isometric KE contractions to further pre-condition the tendon and ensure the participant could generate KE torque at the required loading rate. A 10-cm wide (10–15 MHz) linear probe (Mylab70, Esaote Biomedica, Genoa, Italy) was then positioned sagitally over the tendon (image depth set to 7 cm), which enabled the whole tendon length to be captured in a single sagittal image/movie. Elongation of the tendon during a KE RMVC was measured in each video frame as the displacement of both the patella apex and the tibial tuberosity. The RMVC lasted for 6 s, followed immediately by a 6 s ramped relaxation to rest. At least two RMVCs were performed with 2-min rest in between attempts and, generally, a successful RMVC (and movie) was achieved within two attempts. The loading rate during the 6 s RMVC was COL: 33.6 ± 5.7 Nm s^−1^ vs. PLA: 29.0 ± 9.4 Nm s^−1^ (pre-training) and COL: 32.8 ± 4.9 Nm s^−1^ vs. PLA: 31.7 ± 6.9 Nm s^−1^ (post-training). As the loading rate depended on the participant’s ability to produce maximal voluntary torque, real-time torque-time data were projected in front of the participant, so they could gradually and consistently increase torque output to MVC. After filtering the EMG signal (see above), the torque and EMG data in the AcqKnowledge file were resampled at 23 Hz offline to be synchronized with the ultrasound video, which was sampled at 23 Hz. The administration of a square wave pulse, which was visible simultaneously on the AcqKnowledge software and the ultrasound video *via* the ECG signal was used to synchronize torque and EMG data with the ultrasound video.

#### Vastus lateralis and its subcutaneous adipose tissue (SAT) thickness

With the participant in the anatomical position, femur length (from the mid-point of the greater trochanter to the lateral femoral epicondyle) was assessed with a measuring tape and, at 50% of this distance, the medio-lateral borders of the VL muscle were determined with ultrasound and the distance between them was assessed with a measuring tape. The point corresponding to 50% femur length and 50% VL width was marked on the skin with a permanent marker pen. With the participant seated on the IKD in the resting state (knee and hip joint angles set at 90° and 85°, respectively), the 10-cm wide (10–15 MHz) linear probe (Mylab70, Esaote Biomedica, Genoa, Italy) was placed on this point in the same orientation of the VL muscle fascicles to obtain a sagittal image of the VL. Minimal pressure was exerted on the transducer to prevent compression of the muscle tissue and SAT. VL and SAT thicknesses were subsequently analysed using freely available image analysis software (ImageJ V.1.8.0, National Institute of Health, MD, United States). VL thickness (the perpendicular distance between the superficial and deep aponeuroses) and SAT thickness (the perpendicular distance between the lower border of dermis and the upper border of the superficial VL aponeurosis) were measured as an average of three sites (i.e., 25%, 50%, and 75% of the 10-cm wide image).

#### Analysis of tendon data

Using semi-automated tracking software (Tracker, version 6.0.10, https://physlets.org/tracker/), an average of 335 ± 44 frames during RMVC and relaxation were tracked to measure the displacement of both the patellar apex and tibial tuberosity. Individual tendon force–elongation data were subsequently fitted with a second-order polynomial (*R* ^2^ > 0.90 in all cases). An example of force-elongation data is in Supplementary Data. Patellar tendon mechanical and material properties pre- and post-training were calculated using the weakest maximum absolute tendon force for each participant, usually determined during pre-training testing. Patellar tendon strain was defined as tendon elongation expressed as a percentage of the tendon’s original length, i.e., 100 × change in tendon length (ΔL)/resting tendon length (L_0_). Tendon stress was defined as the peak tendon force (*F*_t_) at KE RMVC relative to the mean tendon CSA (i.e., *F*_t_/CSA). Tendon stiffness (Δ*F*_t_/ΔL) was calculated from the participant’s final 20% *F*_t_ interval obtained from the fitted data. Young’s modulus (E) was calculated by multiplying stiffness (k) with the ratio of the resting tendon length to mean tendon CSA (i.e., E = k × (L_0_/CSA)). Tendon hysteresis was calculated as the difference between the area under the curves regarding the RMVC and relaxation phases and presented as a percentage. An example of force-elongation curve, including both the RMVC and relaxation phases, is provided in Supplementary Figure S1.

#### Test-retest reproducibility of morphological, mechanical and material tendon properties

Test–retest reproducibility of key measurements was determined on eight healthy young men (age: 24.1 ± 4.7 years; height: 1.77 ± 0.05 m; body mass: 73.7 ± 6.1 kg). Participants for this reproducibility test were asked to visit the laboratory twice with a 1-week interval. All measurements were performed by the same researcher (J.L.) and using the same methods, as described above. Coefficient of variation (CV), typical error, and intraclass correlation coefficient (ICC) with 95% confidence intervals (CIs) were used to express inter-day reproducibility (Table 3). For all variables, CVs were low except for tendon hysteresis (14%) and ICCs were high (>0.90) with narrow 95% CIs (0.62–0.99) except for tendon hysteresis (0.54–0.98).

**TABLE 3:** Test-retest reproducibility of morphological, mechanical and material tendon properties.

Variable	CV (%)	Typical error (95% CI)	ICC (95% CI)
PT length (mm)	1.4	0.654 (0.432–1.330)	0.986 (0.924–0.998)
Mean CSA (mm^2^)	1.6	1.566 (1.035–3.187)	0.984 (0.912–0.997)
Tendon force (N)	3.9	154 (102–314)	0.991 (0.950–0.998)
Loading rate (Nm·s^−1^)	8.0	3.516 (2.325–7.157)	0.951 (0.745–0.991)
Stiffness (N·mm^−1^)	5.6	131 (86–266)	0.964 (0.806–0.994)
Young’s modulus (GPa)	5.0	0.057 (0.038–0.117)	0.921 (0.618–0.986)
Stress (MPa)	4.1	1.771 (1.171–3.605)	0.980 (0.889–0.996)
Elongation (mm)	2.5	0.103 (0.068–0.210)	0.965 (0.815–0.994)
Strain (%)	4.2	0.354 (0.234–0.721)	0.966 (0.819–0.994)
Hysteresis (%)	14.0	2.03 (1.343–4.134)	0.902 (0.543–0.982)

### Statistical analyses

All data are presented as means ± standard deviations (SD). Pre-training between group (COL vs. PLA) comparisons of physical characteristics, dietary behavior and total training load during pitch-based sessions were performed with independent *t*-tests. Two-way mixed ANOVA models (group: COL vs. PLA; time: pre-*vs.* post-training) were performed to detect changes in KE and KF MVC torque, resting tendon length, loading rate, mean tendon CSA, all other tendon mechanical and material properties, and thickness of the VL and SAT. When significant group × time interaction effects were found, post-hoc paired *t*-tests (pre-*vs.* post-training for COL and PLA) were performed to reveal between-group differences. A three-way mixed ANOVA was performed to assess differences among group (COL vs. PLA), time (pre-*vs.* post-training), and location (25% vs. 50% vs. 75% tendon length) for tendon CSA. Two effect sizes, Cohen’s *d* (for *t*-tests) and the partial eta squared, η_p_^2^, (for ANOVA interaction) were reported for each statistical model. The thresholds of Cohen’s *d* and η_p_^2^ are defined as small (*d* = 0.20 and η_p_^2^ = 0.01), medium (*d* = 0.50 and η_p_^2^ = 0.06) and large (*d* = 0.80 and η_p_^2^ = 0.14) (Cohen, 1988). Data were analyzed by using the statistical software package SPSS (version 26, SPSS Inc. Chicago, IL) and level of significance was set at *p* < 0.05.

## Results

### Group characteristics

Age, body mass, height and baseline isometric and concentric KE and isometric KF MVC did not differ between COL and PLA groups (all *p* > 0.05, Table 1). All participants were “normally” menstruating women except for two participants (one in COL and one in PLA), who had menstrual irregularity (thus, their menstrual cycle phases could not be estimated). One participant in COL was using the combined oral-contraceptive pill (OCP, Yasmin^®^, 21 days) and had been doing so for 6 months. Due to the challenges involved with testing during in-season (i.e., very limited player availability), it was not possible to schedule the pre- and post-training tests in the same menstrual cycle phase but we recorded when participants were tested according to their phase (Supplementary Table S4).

### Macro- and micronutrient intake

Habitual and total macronutrient and vitamin C intake did not differ between COL and PLA (all *p* > 0.05, Table 2) except for total protein intake (including the COL supplementation).

### Training load during pitch-based session

Total distance, and running and sprinting distance did not differ between COL and PLA (all *p* > 0.05, Supplementary Table S2).

### Maximum strength

Isometric and concentric KE MVC, isometric KF MVC, antagonist co-activation, VL muscle and SAT thickness before and after training are presented in Table 4. There were no main effects for training, group, or interaction effects for any of the variables (*p* > 0.05).

**TABLE 4:** Knee extension (KE) and knee flexion (KF) isometric (iso) and concentric (con) maximal voluntary contraction (MVC) torque, antagonist muscle co-activation and *vastus lateralis* (VL) muscle and subcutaneous adipose tissue (SAT) thickness in COL and PLA groups before (PRE) and after (POST) training. Data are mean ± SD.

Variable	COL (*n* = 8)			PLA (*n* = 9)			
	PRE	POST	DELTA	PRE	POST	DELTA	g × t, *p*
Iso KE MVC (N∙m)	175 ± 45	192 ± 31	16.8 ± 28.1	182 ± 50	184 ± 29.6	2.2 ± 31.3	0.325
Iso KF MVC (N∙m)	84.2 ± 22.0	79.8 ± 20.4	−4.4 ± 14.3	80.9 ± 20.2	77.6 ± 19.3	−3.3 ± 12.4	0.863
Con KE MVC (N∙m)	138 ± 31	139 ± 30	1.1 ± 12.3	132 ± 29	141 ± 22	9.6 ± 26.1	0.849
Ant co-activation (%)	19.6 ± 9.0	18.2 ± 7.8	−1.4 ± 8.3	18.9 ± 9.7	18.3 ± 5.8	−0.6 ± 8.4	0.858
VL thickness (mm)	25.2 ± 2.7	25.7 ± 3.0	0.44 ± 2.15	24.7 ± 2.9	24.9 ± 2.4	0.18 ± 1.31	0.781
SAT thickness (mm)	7.7 ± 2.9	7.4 ± 3.0	−0.07 ± 0.73	6.0 ± 1.6	6.0 ± 2.0	−0.03 ± 0.48	0.887

### Morphological, mechanical, and material tendon properties

Patellar tendon length, mean CSA, force, stress, elongation, strain, and hysteresis data are presented in Table 5. There were no main effects for training or group, or interaction effects for resting patellar tendon length, tendon force and loading rate (*p* > 0.05; Table 5). Regarding mean PT CSA, there was no main effect of training (*F*_1,15_ = 4.290, *p* = 0.056, η_p_^2^ = 0.222), group (*F*_1,15_ = 0.037, *p* = 0.851, η_p_^2^ = 0.002), or training × group interaction (*F*_1,15_ = 3.418, *p* = 0.084, η_p_^2^ = 0.186; Table 5). A three-way ANOVA revealed no training × group × location interaction (*F*_2,30_ = 0.067, *p* = 0.933, η_p_^2^ = 0.004).

**TABLE 5:** Patellar tendon properties in PLA and COL before (PRE) and after (POST) training. Data are mean ± SD.

Variable	COL (*n* = 8)		PLA (*n* = 9)		
	PRE	POST	PRE	POST	g × t, *p*
Resting tendon length (mm)	46.5 ± 5.0	46.4 ± 5.4	43.5 ± 5.7	43.5 ± 5.6	0.610
Mean CSA (mm^2^)	76.9 ± 7.1	77.1 ± 7.2	77.7 ± 8.1	77.7 ± 8.2	0.084
Tendon force (N)	5017 ± 871	5029 ± 776	4680 ± 1038	4369 ± 629	0.356
Stress (MPa)	63.3 ± 9.3	63.2 ± 9.1	55.4 ± 8.1	55.3 ± 8.1	0.134
Elongation (mm)	4.0 ± 1.1	3.4 ± 0.5	3.5 ± 0.8	3.4 ± 0.9	0.275
Strain (%)	8.9 ± 3.0	7.3 ± 1.1	8.0 ± 1.5	8.1 ± 2.5	0.133
Hysteresis (%)	35.1 ± 25.3	37.6 ± 12.6	36.7 ± 14.9	28.2 ± 12.0	0.897

### Tendon stiffness, Young’s modulus and hysteresis

**FIGURE 1:**
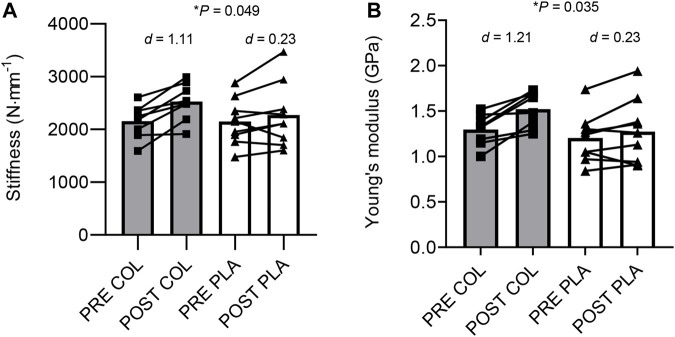
Changes in tendon stiffness **(A)** and Young’s modulus **(B)** in collagen (COL) and placebo (PLA) groups before (PRE) and after (POST) training. *d* = Cohen’s *d*; *Significant group × time interaction effect.

Regarding stiffness at 80%–100% RMVC, there was a main effect of training (*F*_1,15_ = 18.040, *p* < 0.001, η_p_^2^ = 0.546) and a training × group interaction (*F*_1,15_ = 4.589, *p* = 0.049, η_p_^2^ = 0.234) but no main effect of group. Post-hoc paired *t*-tests revealed that post-training stiffness was greater than pre-training in COL (*p* = 0.002) but not in PLA (*p* = 0.186; Figure 1). Regarding Young’s modulus, there was a main effect of training (*F*_1,15_ = 19.916, *p* < 0.001, η_p_^2^ = 0.570) and a training × group interaction (*F*_1,15_ = 5.379, *p* = 0.035, η_p_^2^ = 0.264; Figure 1). Post-hoc paired *t*-tests revealed that post-training Young’s modulus was greater than pre-training in COL (*p* = 0.003) but not in PLA (*p* = 0.142). Regarding hysteresis, there was no main effect of training (*F*_1,15_ = 4.004, *p* = 0.064, η_p_^2^ = 0.211), no main effect of group (*F*_1,15_ = 0.025, *p* = 0.877, η_p_^2^ = 0.002) and no training × group interaction (*F*_1,15_ = 0.017, *p* = 0.897, η_p_^2^ = 0.001).

## Discussion

The aim of this study was to investigate the effect of collagen supplementation on the changes in patellar tendon morphological, mechanical, and material properties after 10 weeks’ in-season soccer training (which incorporated lower-limb bodyweight strength/plyometric training) in the U21 squad of a FA Women’s Super League soccer club. The main findings were greater increases in tendon stiffness and Young’s modulus in the players who consumed collagen hydrolysate supplementation with their soccer training compared to those players who received placebo.

To our knowledge, this is the first study to investigate the changes in patellar tendon properties in high-level female soccer players following a period of training with or without collagen supplementation. We observed no changes in tendon CSA, which suggests the 18% increase in stiffness was explained predominantly by changes in the tendon’s material properties, which is supported by the 17% increase in Young’s modulus. These greater changes in tendon stiffness and Young’s modulus in COL vs. PLA, may be due to augmented mechanical loading-induced tendon collagen synthesis in the presence of high serum concentrations of the necessary exogenous amino acids (i.e., glycine, proline, and hydroxyproline) and vitamin C for synthesizing collagen (Shaw et al., 2017; Lis and Baar, 2019). These key amino acids may have increased the concentration of the collagen-specific cross-linking compound, hydroxylysyl pyridinoline, and/or may have increased collagen fibril density, both of which have the potential to increase tendon stiffness (Couppé et al., 2014; Couppé et al., 2021) in the absence of tendon hypertrophy. Further, orally administered collagen is absorbed within the connective tissue of rodents (Oesser et al., 1999; Watanabe-Kamiyama et al., 2010), while arginine and glycine intake for 7 days increases the amount of hydroxyproline deposition, which indirectly indicates increased collagen synthesis in wounded rat muscle (Chyun and Griminger, 1984). Also, 5 g collagen intake for 12 months in postmenopausal women increased plasma P1NP compared to baseline (König et al., 2018), which suggests that long-term chronic collagen supplementation augments tissue collagen synthesis. When repeated mechanical loading and 20 g collagen supplementation are combined, Lis et al. (2021)) found that lower limb rate of force development (RFD) in healthy male athletes improved. Although tendon stiffness was not directly measured in the study by Lis et al. (2021), muscle-tendon stiffness is related to RFD (Bojsen-Møller et al., 2005), and a training-induced change in tendon stiffness likely affects the change in RFD (Maffiuletti et al., 2016). Thus, augmenting (loading-induced) tendon collagen synthesis with collagen supplementation may have increased connective tissue stiffness in the study by Lis et al. (2021), leading to improved RFD, which would support our main findings.

Although previous studies using high-intensity resistance training for 9–12 weeks found an increase in patellar tendon CSA at the proximal and distal ends (Kongsgaard et al., 2007; Seynnes et al., 2009), the intensity of training in the current study was probably insufficient to induce tendon hypertrophy. In contrast to our findings, a recent study by Jerger et al. (2022) showed that moderate-to-high intensity strength training (70%–85% single repetition maximum) for 14 weeks with 5 g daily collagen supplementation further increased Achilles tendon CSA compared to training alone in previously untrained, healthy, young men. However, the increase in tendon stiffness after training did not differ between PLA and COL in this study (Jerger et al., 2022). The discrepancies between our findings and those of Jerger et al. (2022) are likely linked to the numerous methodological differences between studies. For example, the intensity and frequency of strength training in the study by Jerger et al. (2022) probably facilitated a greater hypertrophic response (+5% in PLA and +11% in COL). However, given the similar P1NP response to 0 g vs. 5 g vitamin C-enriched gelatine and exercise in the study by Shaw et al. (2017), it is noteworthy that just 5 g collagen (with no co-ingestion of vitamin C) led to more than a two-fold increase in tendon size in the COL group in the study by Jerger et al. (2022), although this may be due to the *daily* consumption of 5 g collagen. Further, as tendon stiffness is influenced by both the material properties (e.g. collagen fibril density and cross-linking) and CSA of the tendon (Heinemeier and Kjaer, 2011), it is also notable that this greater hypertrophic adaptation in COL did not confer greater changes in tendon stiffness. Unfortunately, modulus was not reported in the study by Jerger et al. (2022), so it is not known whether between group differences in material properties may explain these results. The other methodological differences between studies, e.g., collagen dose, timing of ingestion, participants’ training history, participants’ sex [e.g. compared to men’s tendons, women’s tendons show a lower collagen synthesis rate following acute exercise and attenuated hypertrophy following training (Magnusson et al., 2007)], the investigated tendon (Achilles vs. patellar), etc., may explain our contrasting findings. Nevertheless, our study is the first to show that a relatively large dose of hydrolysed collagen (plus vitamin C) in combination with soccer training (incorporating bodyweight strength/plyometric exercises) increases patellar tendon stiffness and modulus in female soccer players more than soccer training alone.

Despite the novelty of our study and the important findings, certain limitations should be considered. The current study did not attempt to assess muscle-tendon properties during the same menstrual cycle phase pre- and post-training due to the club’s and players’ restricted time-frame during the research period. It has been suggested that fluctuating serum estrogen concentration does not affect maximum strength (Dasa et al., 2021), tendon properties (Burgess et al., 2010), or patellar tendon collagen synthesis (Miller et al., 2007) in young, active, eumenorrheic women. However, the limitations in these studies (Hansen 2018) suggest it is still unclear whether menstrual cycle phase does affect these variables, while physiologically high estrogen does appear to increase joint laxity (Shultz et al., 2006), possibly by decreasing ligament stiffness. However, whether this would be sufficient to modify tendon stiffness remains to be determined. While future studies should attempt to conduct pre- and post-testing in the same menstrual cycle phase (ideally determined using an ovulation test), this is not practically feasible in elite athletes, given their extremely limited availability. It is also not known whether the inclusion of the single participant using the oral contraceptive pill (OCP) had any impact on our results. Hansen et al. (2009) reported that patellar tendon collagen synthesis and P1NP is lower in OCP users than in non-OCP users. However, the lower serum estrogen concentration in OCP-users does not appear to affect tendon properties (Hansen et al., 2013; Hicks et al., 2017) and, given the fact that only one participant used OCP in our study, this is unlikely to have affected our results. Furthermore, our study incorporated bodyweight strength and plyometric exercises (part of the athletes’ habitual soccer training), while high-intensity strength training may have induced greater tendon adaptation, particularly with regards to tendon hypertrophy. Future studies may therefore wish to implement this mode of chronic exercise to test the hypothesis that COL in conjunction with *high intensity* strength training also improves tendon properties more than strength training alone in female athletes. Although the lead researcher was not blinded to participant group allocation, the participants themselves and the strength and conditioning coaches responsible for the training programs were blinded. Furthermore, in contrast to the manual method of calculating tendon force-elongation curves (used to measure tendon stiffness, Young’s modulus, strain and hysteresis), the semi-automated tracking software used in this study removed significant subjectivity from the data analysis and risk of bias from the lead researcher. The lack of bias is reflected in the lack of group × time interaction regarding MVC force, tendon loading rate and tendon CSA. It should also be noted that tendon stiffness and modulus (the only variables to demonstrate a significant group × time interaction) were calculated using the last 20% of each participant’s *lowest absolute* MVC force (usually recorded at baseline), and MVC force did not differ between COL and PLA at baseline. Thus, it is highly unlikely that our single-blind study design had any impact on the comparative outcome of our data. Finally, although our test-retest data demonstrate high reproducibility for the main measurements used in this study, these assessments were performed in healthy young men, who have greater knee extensor strength and patellar tendon stiffness than age-matched women (Hicks et al., 2013).

In conclusion, 10 weeks’ in-season soccer training (incorporating bodyweight lower-limb strength and plyometric training), supplemented with 30 g collagen hydrolysate (and 500 mg vitamin C) three times a week, conferred greater gains in patellar tendon stiffness and Young’s modulus compared to soccer training alone in a FA Women’s Super League Under 21s squad. These novel findings have significant implications for sport science support in female soccer players. Future studies should investigate if collagen supplementation can improve specific aspects of female soccer performance requiring rapid transference of force, and if it can help mitigate injury risk in this under-researched population.

## Data Availability

The datasets presented in this study can be found in online repositories. The names of the repository/repositories and accession number(s) can be found in the article/Supplementary Material.
