# Supplementary material for: Collagen supplementation augments changes in patellar tendon properties in female soccer players
Source: Front Physiol. 2023 Jan 26;14:1089971. doi: 10.3389/fphys.2023.1089971 (PMC9910607; doi:10.3389/fphys.2023.1089971)
Supplement: Supplementary file 1 [file Table1.docx]

**Supplementary Table 1.** Bodyweight strength and plyometric exercises.

| Bodyweight strength exercise | | | | | | |
| --- | --- | --- | --- | --- | --- | --- |
| Exercise | Volume | Week  1 − 2 | Week  3 − 4 | Week  5 – 6 | Week  7 − 8 | Week  9 − 10 |
| Isometric split squat  (10-s hold) | Set | 3 | 3 | 4 | 4 | 5 |
|  | Repetitions | 3 | 3 | 3 | 3 | 3 |
| Reverse Plank alternative heel raise  (3-s hold) | Set | 3 | 3 | 3 | 3 | 3 |
|  | Repetitions | 8 | 8 | 10 | 10 | 12 |
| Banded sissy squat | Set | 3 | 3 | 3 | 3 | 3 |
|  | Repetitions | 6 | 6 | 8 | 8 | 10 |
| Countermovement jump | Set | 3 | 3 | 3 | 3 | 3 |
|  | Repetitions | 3 | 3 | 4 | 4 | 5 |
| Reverse Nordic | Set | 3 | 3 | 3 | 3 | 3 |
|  | Repetitions | 3 | 3 | 4 | 4 | 5 |
| Nordic hamstring exercise | Set | 3 | 3 | 3 | 3 | 3 |
|  | Repetitions | 2 | 2 | 3 | 3 | 3 |
| Plyometric exercise | | | | | | |
| Broad jump | Set | 3 | 3 | 3 | 3 | 3 |
|  | Repetitions | 4 | 5 | 6 | 6 | 6 |
| Pogo hurdle hop | Set | 3 | 3 | 3 | 3 | 3 |
|  | Repetitions | 2 | 3 | 4 | 4 | 4 |
| Lateral countermovement jump | Set | 3 | 3 | 3 | 3 | 3 |
|  | Repetitions | 3 | 3 | 4 | 4 | 4 |
| Single leg multi direction hop | Set | 3 | 3 | 3 | 3 | 3 |
|  | Repetitions | 2 | 3 | 4 | 4 | 4 |
| Banded acceleration 5 steps | Set | 3 | 3 | 3 | 3 | 3 |
|  | Repetitions | 2 | 3 | 4 | 4 | 4 |
| Fall start acceleration 10 m | Set | 2 | 2 | 2 | 2 | 2 |
|  | Repetitions | 2 | 3 | 4 | 4 | 4 |
