# Supplementary material for: Collagen supplementation augments changes in patellar tendon properties in female soccer players
Source: Front Physiol. 2023 Jan 26;14:1089971. doi: 10.3389/fphys.2023.1089971 (PMC9910607; doi:10.3389/fphys.2023.1089971)
Supplement: Supplementary file 2 [file Table2.docx]

| Variable | Collagen | Placebo | *P* |
| --- | --- | --- | --- |
| Distance covered |  |  |  |
| Total (km) | 143 ± 11 | 149 ± 15 | 0.449 |
| Running (km) | 28 ± 4 | 30 ± 5 | 0.383 |
| Sprinting (km) | 5 ± 2 | 5 ± 2 | 0.622 |

**Supplementary Table 2**. Training load during pitch-based sessions for 10 weeks in collagen and placebo groups.
