# Supplementary material for: Collagen supplementation augments changes in patellar tendon properties in female soccer players
Source: Front Physiol. 2023 Jan 26;14:1089971. doi: 10.3389/fphys.2023.1089971 (PMC9910607; doi:10.3389/fphys.2023.1089971)
Supplement: Supplementary file 3 [file Table3.docx]

**Supplementary Table 3.** The number of nutritional supplements participants had with training sessions or match.

| Session type | COL (*n* = 8) | PLA (*n* = 9) |
| --- | --- | --- |
| PBS | 11 ± 1 | 12 ± 1 |
| PBS and BWSE | 8 ± 0 | 8 ± 1 |
| PBS and PLY | 7 ± 1 | 8 ± 1 |
| Match | 4 ± 2 | 2 ± 1 |

Data are mean ± SD. *PBS*, pitch-based session; *BWSE*, bodyweight strength exercise; *PLY*, plyometric exercise.
