# Supplementary material for: Collagen supplementation augments changes in patellar tendon properties in female soccer players
Source: Front Physiol. 2023 Jan 26;14:1089971. doi: 10.3389/fphys.2023.1089971 (PMC9910607; doi:10.3389/fphys.2023.1089971)
Supplement: Supplementary file 4 [file Table4.docx]

**Supplementary Table 4.** Pre- and post-training tests performed during different phases of the menstrual cycle.

| **Menstrual cycle phase** | | | | |
| --- | --- | --- | --- | --- |
|  | Early follicular | Late follicular | Early luteal | Late luteal |
| PRE  COL (*n* = 6)  PLA (*n* = 8) | 2  2 | 3  3 | 1  2 | 0  1 |
| POST  COL (*n* = 6)  PLA (*n* = 8) | 1  3 | 1  3 | 4  0 | 0  2 |
