# Supplementary material for: Collagen supplementation augments changes in patellar tendon properties in female soccer players
Source: Front Physiol. 2023 Jan 26;14:1089971. doi: 10.3389/fphys.2023.1089971 (PMC9910607; doi:10.3389/fphys.2023.1089971)
Supplement: Supplementary file 6 [file Image1.pdf]

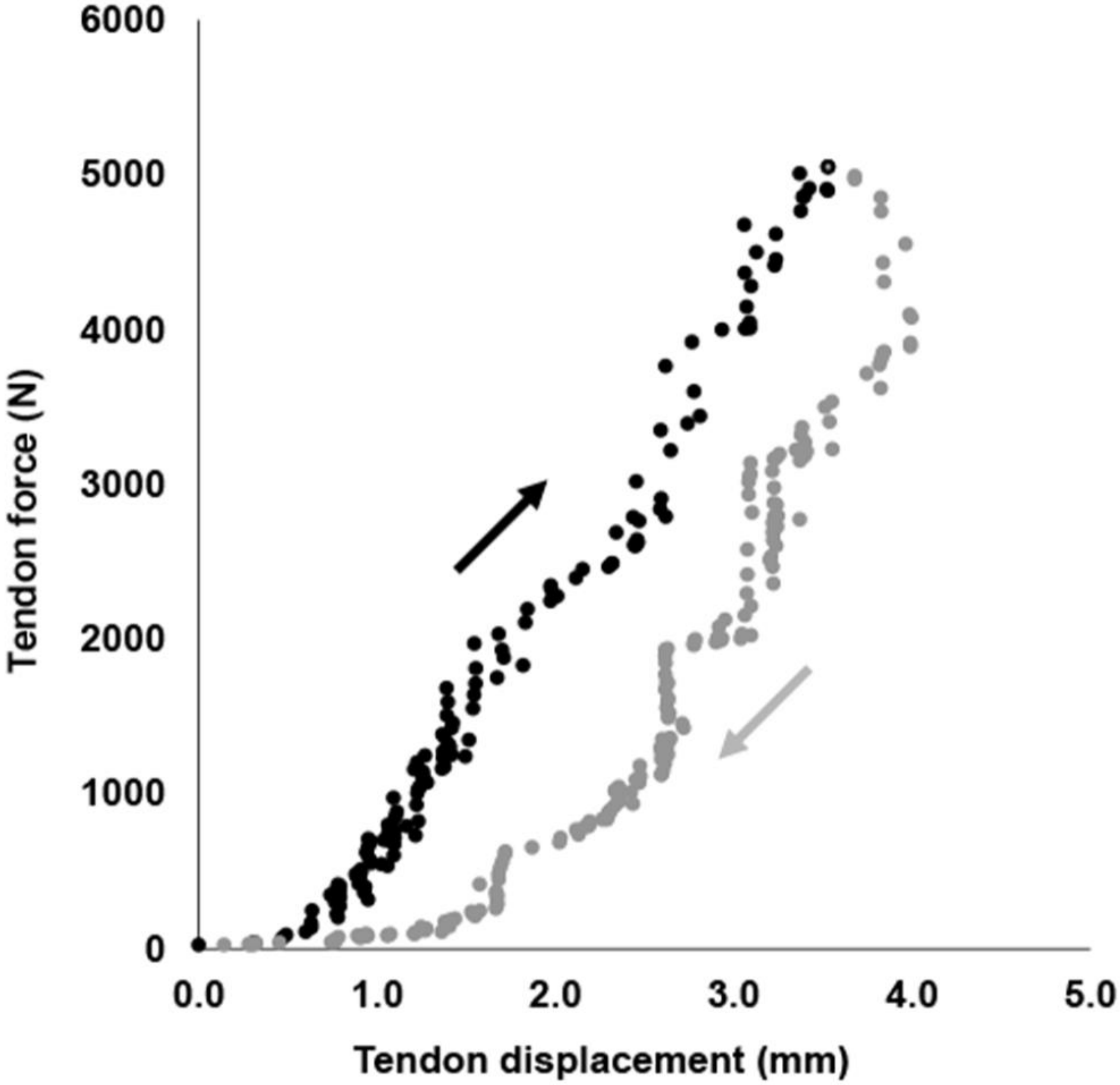

Supplementary Figure 1. An example of the force-elongation curve during a 6-second ramped maximal voluntary contraction (RMVC). Black dots (•) indicate the RMVC phase, while grey dots (•) indicate the relaxation phase.
